# Using Principles from Applied Behaviour Analysis to Address an Undesired Behaviour: Functional Analysis and Treatment of Jumping Up in Companion Dogs

**DOI:** 10.3390/ani9121091

**Published:** 2019-12-06

**Authors:** Nicole Pfaller-Sadovsky, Gareth Arnott, Camilo Hurtado-Parrado

**Affiliations:** 1 School of Biological Sciences, Queen’s University Belfast, Belfast BT9 5DL, UK; g.arnott@qub.ac.uk; 2 Department of Psychology, Troy University, Troy, AL 36082, USA; hhurtadoparrado@troy.edu; 3 Faculty of Psychology, Fundación Universitaria Konrad Lorenz, Bogotá 110231, Colombia

**Keywords:** companion dogs, functional analysis, noncontingent reinforcement, behavioural skills training, ABA

## Abstract

**Simple Summary:**

This study investigated the effects of behaviour-change procedures typically applied with human learners on an often-reported undesired behaviour of companion dogs, i.e., jumping up on people. It was found that jumping up was maintained by owner provided consequences (i.e., access to a preferred object or attention). During treatment, dog owners were successfully taught to implement a time-based reinforcement strategy with high fidelity, which yielded important reductions in jumping up. These findings may be helpful for owners and animal behaviourists alike when assessing and treating undesired behaviours in companion dogs.

**Abstract:**

The aim of this study was to investigate the feasibility and effectiveness of procedures successfully used in human related applied behaviour analysis practices to the field of clinical animal behaviour. Experiment 1 involved functional analyses to identify the reinforcement contingencies maintaining jumping up behaviour in five dogs. Experiment 2 comprised teaching dog owners a noncontingent reinforcement intervention (i.e., time-based reinforcement) via behavioural skills training. Single-case experimental methods were implemented in both experiments. The results of Experiment 1 showed that access to a tangible (dogs D01, D02, D03, and D04) and owner attention (dog D05) were reliably maintaining the jumping up behaviour. Experiment 2 demonstrated that noncontingent reinforcement effectively reduced jumping in three out of four dogs (Tau −0.59, CI 90% [−1–0.15], *p* = 0.026, Tau −1, CI 90% [−1–−0.55], *p* = 0.0003, and Tau −0.32, CI 90% [−0.76–0.11], *p* = 0.22 for dyads D01, D02, and D05, respectively), and that behavioural skills training was successful in teaching owners to perform a dog training intervention with high fidelity. Although the results are promising, more canine-related research into functional analysis and noncontingent reinforcement, as well as implementation of behavioural skills training with animal caregivers, is needed.

## 1. Introduction

Canine behaviour problems (e.g., aggressive responses, destructiveness, stereotypic behaviours; [1,2]) have been found to be the main reasons why owners relinquish their dogs to shelters [3,4,5]. Prior to the breakdown of the human–dog relationship and subsequent relinquishment, owners often try to alleviate their dog’s behaviour problem either on their own or with the help of a professional, such as a certified animal behaviourist, veterinary behaviourist, or certified dog trainer [6]. The focus of typical animal behaviour change approaches relies predominantly on the application of ethological knowledge (e.g., communicative signals) and learning theory (e.g., classical conditioning and the four quadrants of operant conditioning; e.g., [7,8,9]). While the latter are part of Applied Behaviour Analysis (ABA), ABA comprises more than the four quadrants of behaviour (e.g., function-based approach; [10,11,12]).

ABA is the discipline in which tactics derived from the principles of learning and behaviour are applied to improve socially significant behaviour, and experimentation is used to identify the variables responsible for the improvement in behaviour [13]. Although ABA has proven highly useful in the human behaviour change arena for addressing a number of undesired behaviours (e.g., aggressive responses, anxiety, destructiveness, pica, self-injurious behaviour, stereotypic behaviour; [10,14]), the integration of ABA into the applied and/or clinical animal behaviour setting is still in its early stages [15,16,17]. Some of the unique features of ABA that are specifically valuable for animal behaviour professionals in facilitating behaviour change in animals and their caregivers ([18,19]) include (a) a function-based approach to the identification of problematic behaviours and related development of interventions (e.g., functional analysis [FA]; [20]); (b) scientific methods that focus on continuous data collection of the individual’s behaviour to implement and evaluate progress in an evidence-based approach (single-case research methods; [SCRMs]; [16,21]); (c) emphasis on working with owners and caregivers in the settings where the problematic behaviours occur; and (d) focus on humans’ and animals’ wellbeing in the decision-making process.

Adding to previous applied animal behaviour analytic research (e.g., [18,22,23,24,25,26,27]), the current study demonstrates how ABA can make a comprehensive contribution to the treatment of canine behaviour problems.

### 1.1. Introduction to Applied Behaviour Analysis

It is useful to consider the defining characteristics of ABA as pillars [28]. Although they are presented separately, it should be noted that the components they represent are closely intertwined.

The first foundational pillar comprises a unique research methodology—single-case research methods (SCRMs) [29]. Research in the experimental and applied analysis of behaviour has extensively used inductive methods of investigation, in which many data points are collected on a few participants with the aim of assessing causal relations between environmental events and behaviour [16]. Behaviour-analytic research carefully identifies and defines target behaviours, assesses interobserver agreement and takes steps to minimize the influence of sources of bias, while maximising experimental control [21]. This is typically achieved by the use of SCRMs which focus on questions involving the application of behaviour-change interventions to individuals rather than questions about population-level patterns [16,30]. SCRMs should be contrasted from case studies which are often unsystematic, subjective, and qualitative in nature [16,21]. Key features of SCRMs include baseline assessment, continuous measurement, stability of performance, and use of different phases [31]. Continuous measurement has clinical relevance as it enables practitioners to observe specific aspects of an individual’s behaviour repeatedly over the course of multiple phases [16]. There are many variations of SCRMs (e.g., reversal design, multi-element design, multiple baseline across behaviours, participants or settings, or changing criterion design), and each design can have its individual characteristics and uses [13]. Comprehensive descriptions of the various types of SCRMs can be found elsewhere (e.g., [16,28,32]).

The second pillar is the contingency of reinforcement. A contingency of reinforcement is the relationship between the occurrence of a response and the consequences that follow and maintain the behaviour [33]. The smallest unit of behaviour (B) and consequence (C) relationship is called a two-term contingency (i.e., B→C). However, contingencies of reinforcement can comprise more than two terms (e.g., introducing a discriminative stimulus; S^D^: B→C), and the effectiveness of reinforcement depends on events called motivating operations (MO; e.g., satiation or deprivation) [34]. Additional to altering momentarily the effectiveness of a given reinforcer (decreasing or increasing effects), MOs simultaneously alter the frequency of the behaviour that has been regulated by that reinforcer [35].

The third pillar comprises investigation of functional relations by implementing “functional behaviour assessments” (FBA). FBA is an umbrella term describing different assessment strategies that are conducted prior to the onset of a given intervention. These aim to identify environmental events (e.g., reinforcement) contributing to the maintenance of problem behaviours [36,37]. FBAs comprise (a) indirect assessments (no direct observation of behaviour, instead questionnaires, rating scales and interviews are implemented); (b) descriptive assessments (direct observation of behaviour without manipulation of antecedents or consequences, using tailored recording methods); and (c) functional analyses (direct observation of behaviour and systematic manipulation of antecedent and consequent events to demonstrate functional relationships between them and behaviour) [36,38].

The fourth underpinning combines a set of various characteristics that are defining features of ABA. These involve: (a) being applied, i.e., that the target behaviour is selected due to its significance to the individual organism, community or society; (b) being behavioural, i.e., that the responses are objectively defined and measurable; (c) being analytic, i.e., that there has been a demonstration that the intervention is responsible for changes in the target behaviour; (d) being technological, i.e., that the procedures allow the possibility of replication; (e) being conceptually systematic, i.e., that research provides descriptions of interventions and changes in behaviour that align with principles in behaviour analysis; and (f) being generalizable, i.e., that outcomes are lasting and occur across different contexts (e.g., environments, people, or times of day) [39,40,41].

The fifth and last pillar is concerned with “social validity”. Social validity is typically measured by questionnaires, surveys, interviews, rating scales or a combination of them [42]. These measures are designed to provide consumers of an intervention (e.g., caregivers, staff, or teachers) with the opportunity to give feedback about how well specific elements of an intervention were liked or disliked [42]. This has clinical relevance, as outcomes of social validity evaluations can have an impact on the decision of practitioners to make changes to the treatment programme. For instance, studies on social validity studies have found that caregivers prefer (a) function-based interventions and (b) interventions that use reinforcement strategies [43,44,45].

### 1.2. ABA and Clinical Animal Behaviour

Despite the long history of ABA’s successful treatment of challenging behaviour in human learners [10,46], ABA has been slowly gaining recognition in the field of clinical animal behaviour. This is surprising in at least two respects: first, a recent study surveyed 130 “animal behavior professionals” (e.g., Certified Applied Animal Behaviorists or Certified Professional Dog Trainers) and Board Certified Behavior Analysts^®^ (BCBA^®^) [47,48]. The purpose of the survey was to investigate commonalities and differences in the professional work of these two populations of behaviour professionals. Assessment and analysis of behaviour were not addressed, however. To summarise, the results demonstrated that few respondents reported using punishment techniques. For example, the majority of animal professionals and BCBAs stated that they predominantly used reinforcement strategies (i.e., 96% and 89%, respectively). Only 1% out of all respondents mentioned that they often used punishment, which underscores the reluctance of both groups of professionals to implement these procedures [48]. This is consistent with current research on the use of punishment procedures in dogs, including the potential detrimental effects (e.g., stress-related behaviours, elevated cortisol levels, or aggression; [49,50,51,52,53]). It is also consistent with ethical guidelines put forward by the BACB^®^ (e.g., Sections 4.08 to 4.10 in the Professional and Ethical Compliance Code for Behavior Analysts; [47,48]), and animal professional accreditation bodies regarding the emphasis on reinforcement procedures in behaviour change programmes (e.g., Least Intrusive, Minimally Aversive Standard; [54,55]). Second, although limited in number, studies have shown that ABA-based interventions are effective in treating problem behaviours in companion animals, as well as teaching caregivers (owners, staff or volunteers) the necessary skills to successfully implement the individual intervention programmes (e.g., [18,22,23,56,57]).

### 1.3. The Current Study

Jumping up on people is an undesired behaviour that companion dogs frequently display [58]. While being a nuisance behaviour for most owners, it can be potentially dangerous to vulnerable individuals, such as small children or elderly adults [58,59]. Various training approaches have been suggested for treating jumping up. For instance, stopping the dog from jumping by stepping on a lead [59], spraying the jumping dog with androstenone-infused water [60], or, more importantly, teaching alternative responses (e.g., sitting or lying on a mat) when a person enters the premises [58,61,62]. However, these training approaches have been put forward in absence of establishing the cause of jumping or, in an ABA context, without conducting a FBA prior to the onset of the intervention. Additionally, the majority of the above cited training approaches require the employment of certain tools (e.g., house lead or spray bottle) and respective skills from the caregivers (e.g., correct timing to step on the lead).

The present study employed procedures often successfully used in human-related ABA practice, namely (a) functional analysis (FA; [20]), (b) noncontingent reinforcement (NCR; [63]), and (c) behavioural skills training (BST; [64,65]). Our overall aim was to exemplify the feasibility and effectiveness of these procedures within the field of applied and clinical animal behaviour.

The FA methodology developed by Iwata et al. [20] used four analogue conditions which were systematically alternated, and each condition manipulated antecedent events and the consequences that followed [41]. For example, in the attention condition, the therapist ignores the individual (occupying him or herself with a typically occurring activity) until the learner displays the target behaviour. The therapist provides attention contingent on the occurrence of the undesired behaviour. The attention condition assesses if the undesired behaviour is maintained by social positive reinforcement [41].

While FAs have been used successfully in identifying the functions of undesired behaviours in dogs (e.g., [24,57,59]), Behavioural Skills Training (BST; an active learning approach in which instruction, modelling, and feedback are sequenced to facilitate skill acquisition; [66,67]) has only been implemented in two animal-related studies [18,68]. Both studies used BST to teach animal caregivers (shelter volunteers and scent detection trainers) the necessary skills to train their respective animals (shelter dogs and pouched rats). In this vein, BST was used in the current study to teach owners the intervention, namely noncontingent reinforcement (NCR).

NCR is described as response-independent or time-based delivery of stimuli with known reinforcing properties [63]. Despite the popularity and the mounting evidence for the ease of implementation and effectiveness with human learners (e.g., [63,69,70,71,72,73]), to the best of our knowledge, there are no published studies using NCR with dogs or other companion animals in applied settings. Thus, the current study takes a novel approach to investigate NCR’s feasibility and efficacy with companion dogs and their owners.

Experiment 1 consisted of a FA—based on a systematic replication of Dorey et al. [59]—which informed the decision on the type and use of reinforcement during the second experiment. Experiment 2 entailed teaching dog owners an NCR procedure by means of BST. Five owner–dog teams (“dyads”) participated in the first experiment. After completing Experiment 1, dyad D04 dropped out due to the owner’s work-related time restraints. The remaining four dyads progressed into Experiment 2.

## 2. General Method

All research was undertaken following approval from The School of Social Sciences, Education and Social Work Ethics Committee, Queen’s University Belfast (ethics application reference number SREC084).

This section reports methodological information which applies for both experiments of this study.

### Participants

Five dogs with owner-reported undesired jumping up behaviour participated in the current study. All five dyads lived in private dwellings (e.g., houses or apartments), and the dogs were kept as typical companion dogs (i.e., “accompanying and associating with one’s dog and the relationship between the owner and the dog that results from such interaction”; [74]). Table 1 provides detailed participants’ information. Based on the information retrieved during the initial screening (questionnaire and interview at the first visit), the owners selected potential reinforcers of jumping up for their respective dog.

If there were more than one owner in a household, the family chose one primary caretaker who participated in the experiments with the dog (D01, D03, and D05). In these cases, all other family members were asked to limit their interactions with the dog to their usual daily routine and were asked to refrain from additional training. In those households that owned multiple companion animals (D01, D03, and D04), the non-participating pets were either briefly moved to a different room or had restricted access to the assessment area during data collection (D03 and D04). These measures were implemented to limit confounding effects during the intervention. In the case of D01, none of these strategies were possible, due to the old age of the second dog (13 years), and the stress that restraining her to a different room might have caused. However, during the screening visit (prior to the initiation of Experiment 1), it was found that the dog did not have any interest in a training-related activity with the participating dog (D01). Hence, it was decided that the second dog could be left unrestrained as she was not interfering with data collection.

## 3. Experiment 1: Functional Analysis (FA)

This experiment aimed to identify the environmental stimuli that reinforce and maintain jumping up behaviour. During the FA, a condition of higher-level jumping-up was identified. The reinforcer that was identified during the previous condition informed the decision regarding which type of reinforcement was to be used during Experiment 2 for each individual dog. Jumping up was defined as the dog lifting both forelegs off the ground while touching the owner’s body above ankle height with any part of the dog’s body.

### 3.1. Method

Based on the information retrieved during the initial screening (questionnaire and interview at the first visit), the owners selected potential reinforcers of jumping up for their respective dog. Those items selected were tangibles that the dyads interacted with on a daily basis. During the screening, owners also reported repeated occurrences of jumping up when holding and/or handling these items. Table 1 provides details on those chosen reinforcers (i.e., edibles and tangibles). Four of the five dyads selected the dogs’ preferred items as reinforcers. Dyad 05, however, chose leashing the dog and briefly leaving the house as tangible reinforcement (same as [59]) and tactile play for the control condition. Each reinforcer was tested in one condition, and each dog was assessed in four conditions and one control condition.

Each condition (attention, control/play, demand, ignore, and tangible) was implemented separately for 3 min in individual sessions (i.e., 3 min constituted one session) until all five conditions were presented (in five consecutive 3 min sessions). The implementation of all five conditions happened in a randomised order (Microsoft^®^ Excel^TM^ random function). Five conditions constituted a cycle. For analysis and display of data, a multi-element design was used (rapid alternation of two or more distinct treatments while their effects on the target behaviour were measured; [13,16]).

The owner opening the door signalled the onset of a condition and data collection started. During the 5 min breaks between conditions, the owner left the assessment area to mark the end of that condition; this provided the antecedent event for the next condition and gave the dog a break. During these periods, the dog stayed in the assessment area with the first author but was ignored (not looking at or talking to the dog).

#### Conditions

During the attention condition, the owner walked into the room and did not pay attention to the dog unless he or she jumped up on the owner. When jumping occurred, the owner delivered tactile and/or vocal attention as was typically done for 10 s. Vocal and/or tactile attention was based on the owners’ typical responses when their dogs jumped up which could be generally labelled as “good natured”, e.g., telling the dog to stay down while gently touching the dog. After 10 s of attention, the dog was ignored until he or she jumped up again. As a consequence of jumping on the owner, the owner delivered attention again for 10 s. This procedure was repeated until 3 min (one session) elapsed and was repeated for each dyad. After 3 min, the session was completed, and the owner left the room.

During the demand condition, the owner presented three requests to the dog. Prior to the onset of data collection (during the screening interview), the owner selected two requests to which the dog already responded to (i.e., requests present in the dog’s repertoire), and one that was new to the dog. Immediately after entering through the front door into the assessment area, these requests were delivered by the owner throughout this condition. If the dog complied with the requests, he or she received a treat. If the dog did not comply with the requests or emitted any other behaviour within 5 s of the demand, the demand was repeated until the dog complied. If the dog jumped on the owner, she stood still, did not look at the dog for 10 s, and let the dog escape from the demand. After 10 s of stillness, the owner gave another request. Table 1 provides details of requests and types of treats used during the demand condition. This condition was the same for each dyad.

During the ignore condition, the owner entered the room but did not pay attention to the dog, i.e., owner did not engage in eye contact, speaking to the dog, or otherwise interacting with the dog, even if the dog jumped up on the owner. This procedure lasted the entire session of the condition and was the same for each dyad.

During the play condition, the dog was given his or her preferred toy at the entrance of the owner. The dog could interact with the toy for the entire session. The owners provided vocal and/or tactile attention every 10 s for the duration of 10 s. Dyads D01, D02, D03, and D04 used toys during this condition (squeaky plush toy, plush toy, fleece blanket, and canvas dumbbell, respectively). However, the play/control condition was conducted differently for dyad D05. Instead of playing with a toy, the dog was offered age-appropriate “rough-and-tumble” play with the owner, interspersed with 10 s intervals of “dog-directed-speech” [75], and 10 s intervals of not talking to the dog but continued play.

During the tangible condition, a different set of items was used (please see Table 1 for respective tangibles). These items were chosen by the owners under the assumption that the respective dog would jump to get access to them. At the onset of the condition, the owner walked through the door holding the object in sight and in a height that the dog could obtain the item if he or she jumped up on the owner. If jumping occurred, the item was given to the dog and he or she could interact with it for 10 s without any dog-directed speech from the owner. After 10 s had elapsed, the owner picked up the object, held it in sight and waited for the next occurrence of jumping up. This sequence was repeated until 3 min had elapsed (one session). This condition was conducted differently for dyad D05. Instead of access to a preferred item, dog D05 was leashed and led out of the front door for approximately 10 s as a consequence for jumping up. After 10 s, the dog was walked into the house and unleashed, after which the front door was closed behind them. The owner waited for the next response, put the lead on and walked a few paces outside. This procedure was repeated for the entire session.

The number of FA cycles (five conditions in randomized order with each condition or session lasting 3 min in duration) presented to each dyad varied, as each dyad could not proceed to the treatment phase (Experiment 2) until a condition with increased responding was identified, as compared to other conditions. Table 2 provides an overview on the number of cycles per dog.

### 3.2. Interobserver Agreement

Two observers independently scored video recordings of the undesired behaviour (occurrences of jumping up). Percent agreement was assessed on a session-by-session basis for more than a third of the recordings. Agreement on occurrences of jumping up was calculated by dividing the number of agreements by the number of agreements plus disagreements and multiplying by 100. Calculation yielded a mean agreement of 96% across 25 sessions (five cycles, one cycle for each dyad, one cycle comprised five sessions) with 100% agreement for dyads 01, 03, 04, and 05 and 80% agreement for dyad 02 (i.e., one disagreement). The cycles were selected by using the Microsoft^®^ Excel^TM^ random function.

### 3.3. Data Analyses

**Figure 1:**
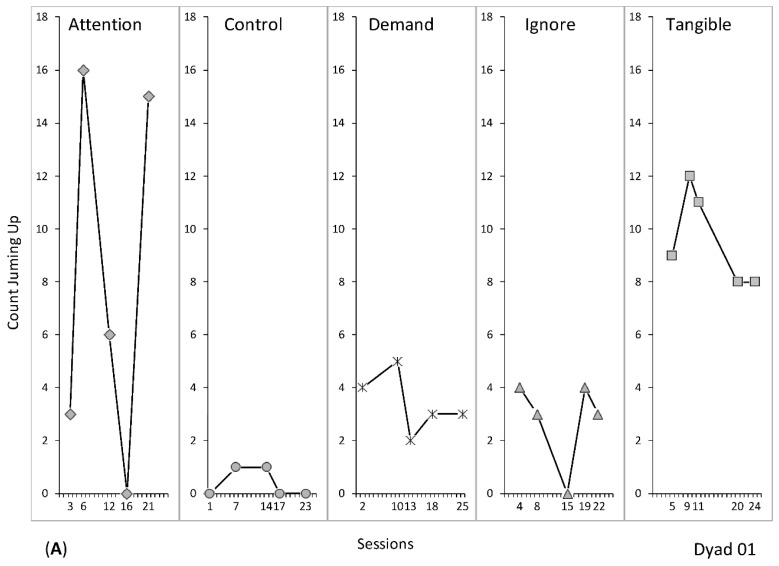

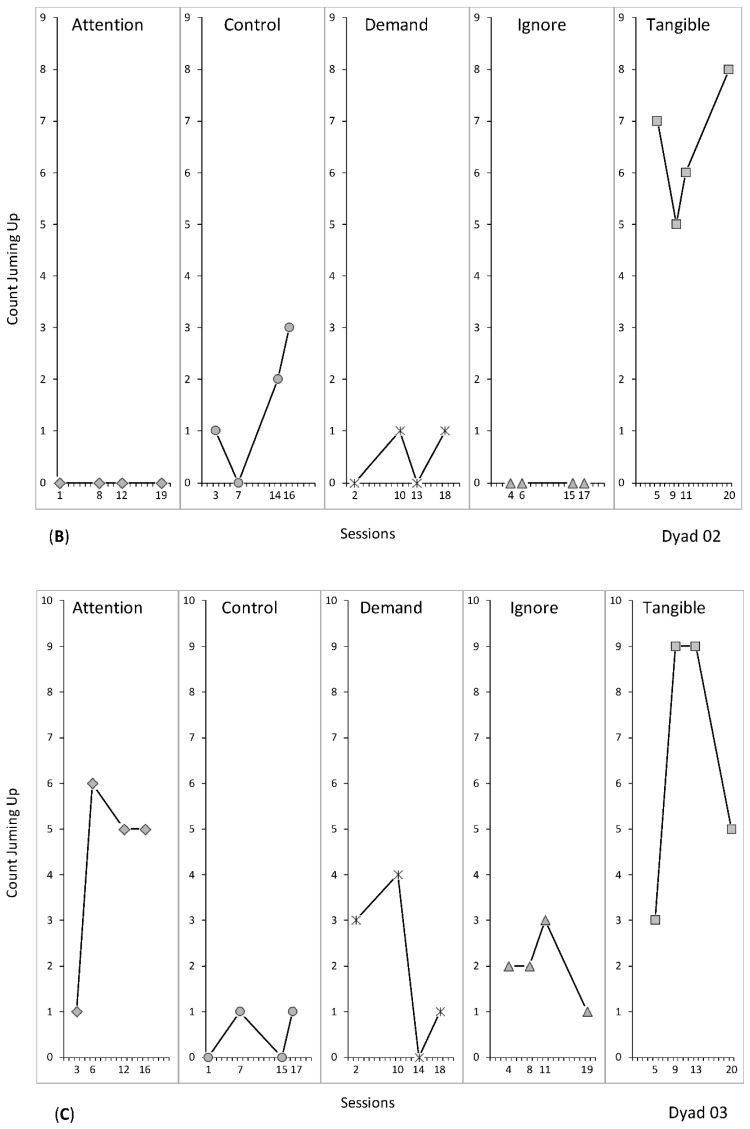

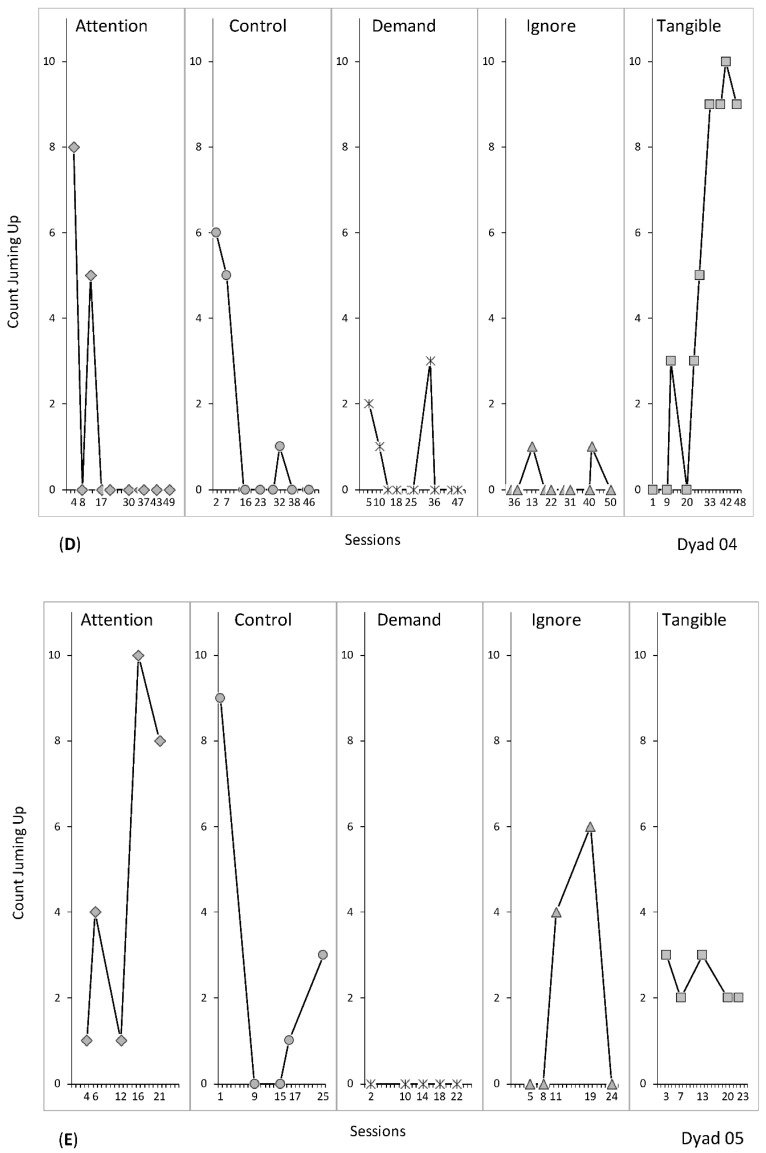
Counts of jumping up behaviour during functional analysis conditions (i.e., attention, control, demand, ignore, and tangible) are shown for (**A**) dyad D01; (**B**) dyad D02; (**C**) dyad D03; (**D**) dyad D04; and (**E**) dyad D05. Each figure displays numbers of weekly cycles (i.e., five sessions; x-axis), and count of jumping up responses (y-axis) for each dyad. The data paths represent the behaviour changes during each condition.

Visual analysis was used to assess level, trend, and variability of data paths (comprising line graphs, Figure 1A–E; [76]). To support visual inspection and to clarify functions of jumping up across all dyads, Tau effect size indices were calculated (as recommended by [77,78]). This was conducted in an effort to determine the magnitude of level differences between single conditions (i.e., attention, control, demand, ignore, and tangible—Experiment 1) and to assess effectiveness of the NCR procedure (Experiment 2). A free online software tool was used for Tau computation [79]. Tau is an effect size index for SCRMs, and it is based on Mann–Whitney U and Kendall’s Rank Correlation [79]. The Tau effect size index is defined as the percent of data nonoverlapping between baseline and intervention phases [80]. For the current analysis, Tau was chosen because it provides *p*-values (a *p* < 0.05 was considered statistically significant) and confidence intervals, and its values are directly interpretable [80,81]. Magnitude of level differences between conditions can be interpreted as higher values representing a larger effect, while lower values represent a weaker effect (e.g., ≤0.60 = weak effect, 0.61 to 0.92 = medium to large effect, and >0.93 = very large effect; [81,82,83,84]). Data for Experiments 1 and 2 can be found here: https://osf.io/3wg9a/ [85]. Tau calculations for Experiment 1 and 2 across all dyads are provided in the Supplementary Materials (Table S1).

### 3.4. Results

Figure 1 displays the results of the functional analyses for all dyads. The data of dog D01 (see Figure 1A) yielded low counts of jumping up during control, demand and ignore conditions, with an overall mean of 6.6 jumps across sessions (range, 0.4 to 3.4 jumps). Levels of jumping up increased in attention and tangible conditions, yielding an overall mean of 17.6 jumps (8 and 9.6 mean jumps in attention and tangible conditions, respectively). During visual analysis of dog D01′s data, it was found that tangible conditions showed slightly higher level and less variability than attention conditions. This suggested maintenance of jumping up by access to the tangible item (Figure 1A). However, to clarify the ambiguity between attention and tangible conditions, a quantitative analysis was warranted (Tau calculations).

Table 3 shows Tau values and corresponding statistical information (*p*-values and confidence intervals-CI) for each condition contrast for dyad D01 (e.g., control vs. attention). Overall, levels of treatment conditions (attention, demand, ignore, and tangible) were above the control condition (Number 1 to 4, Tau’s 0.72 and 1, CI 90% [0.090–1] and [0.370–1], *p* = 0.0601 and 0.009, respectively). However, only contrasts Number 2 and 4 were statistically significant (*p* < 0.05). Put differently, positive Tau values indicated increased responding during contrasted conditions (e.g., tangible as in contrast Number 4, Table 3), while negative Tau values indicated a decrease in responding during a given contrasted condition (e.g., demand as in contrast Number 6, Table 3).

When conditions were further contrasted against each other, the tangible condition yielded the largest differences in level (e.g., Number 8, Tau 1, CI 90% [0.370–1], *p* = 0.009). However, contrast Number 5 (attention vs. tangible) yielded a small effect with a large CI (90% [−0.430–0.830]) and the difference was not statistically significant (*p* = 0.6015). This result confirmed the ambiguity found during visual analysis of attention and tangible conditions. This may suggest that jumping up was multiply controlled by attention and tangible reinforcement. In an effort to further clarify the maintaining reinforcer (subsequently to be used for the treatment of jumping), further analysis was conducted by averaging contrasted conditions according to the attention or tangible condition (via “Weighted” function of the Tau calculator). These results showed that the average effect of the attention condition was weak (Tau −0.1867, CI 90% [−0.5504–0.1771], *p* = 0.3985), while the average effect for the tangible condition was strong and statistically significant (Tau 0.73, CI 90% [0.3696–1], *p* = 0.0009). After conducting this systematic investigation, it was concluded that tangible reinforcement was the maintaining consequence for the jumping up behaviour of dog D01.

Tau calculations supported the functions identified with visual analysis for the remaining dyads as well (i.e., D02, D03, D04, D05). However, only the results for dyad D01 were presented in detail here to illustrate the usefulness of this approach, in particular to resolve the somewhat ambiguous decision between the two conditions at hand (i.e., attention and tangible). Tau calculations for all the dyads are available in Table S1.

Results for dog D02 showed that the jumping up was maintained by access to the tangible item (Figure 1B). While occurrences of jumping up were low in the attention, control, demand, and ignore conditions (overall mean of 2 jumps with a range from 0 to 1.5 jumps), counts of jumping were high from the onset of the tangible condition and remained high with a mean of 6.5 jumps (range of 3 to 9 jumps) across the condition.

Data for dog D03 indicated maintenance of jumping up by access to the tangible item (Figure 1C). Counts of jumping up were low during the control, demand, and ignore conditions, yielding a mean of 0.5 jumps during control, and a mean of 2 jumps during the demand and ignore conditions. While data of the attention condition increased from 1 jump to 6 jumps during the second cycle (session six), tangible condition data started at 3 jumps during the first session and tripled during successive cycles yielding a mean of 6.5 jumps across the tangible condition.

Dog D04 experienced 10 cycles (50 sessions) of FA conditions. This extension was warranted to obtain a conclusive result on the function of jumping up as data of the first six cycles (30 sessions) were inconclusive. Counts of jumping up stabilised at around nine jumps during the successive four cycles (sessions 31 to 50) of the tangible condition. A mean of 4.8 jumps (range, 0 to 10 jumps) across all tangible conditions was found. The resulting data showed that jumping on the owner was maintained by access to the tangible item (Figure 1D).

Data for dog D05 indicated that jumping up was maintained by the owner’s attention (Figure 1E). Jumping up during the attention condition occurred twice as often (mean 4.8 jumps) as during control and tangible conditions (means of 2.6 and 2.4 jumps, respectively).

### 3.5. Discussion

Reinforcers maintaining the behaviour of jumping up were identified through visual analysis of data paths, supplemented with Tau effect size calculations, in all five dyads. For the majority of dogs, access to a tangible item was the maintaining reinforcer. Although visual inspection of dog D01′s data suggested a tangible function of jumping up behaviour, the differences in level between attention and tangible data paths were ambiguous. To confirm potentially reinforcing stimuli for D01, a quantitative analysis using the Tau effect size index was performed [78]. The Tau computation yielded a large, statistically significant average effect for the tangible condition. Therefore, confirming tangible reinforcement as the function of jumping up in dog D01. It has to be noted that inclusion of tangible conditions in FAs with human participants yielded false-positive outcomes [86,87]. Hence, the tangible condition should be implemented with care and include items that are typically found in the participants’ environment [86]. Although data indicated that tangible reinforcement was responsible for an increase in responding, an alternative interpretation might be that a new function was established, which reduced attention’s reinforcing effect, whereas the tangible stimulus increased in value. Only for dog D05, the owner’s attention was maintaining the undesired behaviour. This shows that owner-delivered consequences, such as access to an item or social attention, maintain problematic behaviour (e.g., positive reinforcement).

These findings are in line with previous research on the effectiveness of FAs in dogs [24,57,58]. All of these studies found that FAs can be useful in determining the function of problem behaviours (e.g., jumping, stereotypic behaviour, aggressive responses), irrespective of the setting and the person conducting the assessment. Previously used settings comprised either private dwellings or shelter locations, and functional analyses were either researcher- or owner-conducted or both. In the studies by Dorey et al. [59] and Winslow et al. [24], one of the authors was conducting the FAs, while in Hall et al. [57] both owner and researcher led individual dogs’ assessments. In the current experiment, owners conducted the functional analyses but were guided by the first author by letting them know when a 3 min session was completed, for example.

This is of interest as previous findings in the human-related literature showed that FAs conducted by parents or other caregivers more reliably identified functions of undesired behaviour [88]. These results may point to the notion that involving caregivers, such as dog owners, in conducting FAs may lead to more meaningful outcomes compared to having external individuals (e.g., animal behaviourists) conducting the assessment. Feuerbacher and Wynne [25], for instance, found that owners can be powerful reinforcers for dogs. It therefore follows that by identification of the reinforcers of the target behaviour, it should be possible to manipulate the reinforcement contingencies in question to decrease the behaviour of interest [57]. For example, disruption of the contingency between behaviour and reinforcement should cause the behaviour to decrease (e.g., by using NCR) [73,89,90]. However, more research on conducting FAs with companion animals is needed. Future studies could investigate comparisons of owner-conducted or animal behaviourist-conducted FAs to get a clearer picture of which approach may yield more meaningful outcomes.

## 4. Experiment 2: Behavioural Skills Training and Non-Contingent Reinforcement

The aim of Experiment 2 was to use BST to teach dog owners an NCR strategy that would allow to treat their dogs’ jumping up responses. NCR is well-established as a treatment for various socially mediated problem behaviours in humans [90]. Mounting research has demonstrated that NCR interventions are easy to implement, effective in weakening the target behaviour, and relatively free of side effects often found with other response-decreasing interventions (e.g., [73,91]). Due to the reported effectiveness of NCR with human learners, and the fact that no published studies using NCR as an intervention with companion dogs could be found, NCR was the obvious choice to reduce jumping up behaviour.

The decision on which types of reinforcement were to be used during the NCR intervention was informed by the findings of Experiment 1, enabling tailored reinforcement for each dyad. Owners were instructed by using BST. This is an approach that uses hierarchically intensifying types of instruction (oral instruction, modelling, and modelling and feedback) to determine the relative efficacy of each instruction type [18,68].

### 4.1. Method

#### 4.1.1. Participants

Four dyads (D01, D02, D03, and D05) that participated in Experiment 1 progressed into Experiment 2. The remaining dyad (D04) dropped out after completing Experiment 1 due to the owner’s work-related time restraints.

The interventions were implemented in the same area of participants’ homes, as previously described. To control for extraneous variables (e.g., other members of the household) and to ensure that the environments were as similar as possible across dyads, data collection was limited to the entrance area of the respective dwellings (e.g., foyer). This decision was based on the findings of previous studies (e.g., [58,59]), and anecdotal observations by the first author that companion dogs engage more frequently in jumping on owners when located at the entrance area of their homes, and after a period of separation has elapsed.

#### 4.1.2. Data Collection and Experimental Design

The intervention delivered the previously identified reinforcers (during Experiment 1) on a time-based schedule, irrespective of the dogs’ behaviour (NCR; [63]). If the jumping up response was maintained by either access to a tangible (dyads 01, 02, and 03) or social positive reinforcement (attention; dyad 05), these reinforcers were noncontingently presented by the owner to treat jumping up. The NCR fixed-time schedule comprised reinforcement delivered in three owner-dog interactions per minute, with each interval (interaction/no interaction) lasting 10 s. The five components of the NCR training entailed (a) entering through the front door, with upper body lowered, but standing, with hands held towards the floor; (b) delivering 10 s intervals of interaction (toy or vocal/tactile); (c) ignoring the dog and standing still for 10 s; (d) repeating the previous sequence three times; (e) leaving the training area (foyer) through the front door. A 100% correct implementation (Step 6 in task analysis displayed in Table 4) was recorded when all of the above components were met. The necessary owner skills to implement these components with fidelity were taught via BST.

To identify the most effective approach for owner education, a sequential implementation of increasingly intensifying methods of owner training was evaluated (BST). The experiment consisted of the following phases: (a) baseline, i.e., counts of jumping up during the first minute of each tangible or attention condition of the functional analyses; (b) oral instruction; (c) modelling by the first author; (d) modelling and feedback; (f) generalisation conducted by the first author; and (g) a follow-up probe. Based on the availability of the participating owners, one weekly training session was performed. One session was 1 min in duration. A maximum of six sessions could be conducted for each instructional phase. Table 4 displays the vocal instructions used during oral instruction phase.

##### Dependent Variables

The count of jumping up responses during 1 min sessions was the primary dependent variable. It was measured to investigate the effectiveness of the NCR procedure on jumping.

The percentage of procedural integrity with the NCR procedure was the second dependent variable. It was calculated for owners and the first author (generalisation phase). Procedural integrity was computed as the number of training steps performed (Table 5) divided by the number of possible steps during a session, and then converting this result to a percentage [18].

Table 5 displays the procedural integrity hierarchy of the NCR training approach, which was taught to the owners via BST. The hierarchically ordered list, from fully correct to least correct (Step 6 to 0, respectively), was used to define and quantify owners’ procedural integrity with the NCR dog training procedure.

#### 4.1.3. Design and Procedures

A changing conditions single-case experimental design (i.e., multiple phase changes; [92,93]) was used to evaluate the effectiveness of NCR on behaviour change of the dogs (count of jumping up), and the efficacy of BST phases on owner procedural integrity (percentage). As additional measures for evaluation of effectiveness of the NCR approach, inter-response times (IRT) between jumps, as well as effect sizes (Tau and corresponding statistics) were computed (Table 6).

Data from the FA conditions with the highest occurrences of jumping up (Experiment 1; tangible or attention) were used as initial baseline data. In the first intervention phase (oral instruction, OI), the information about how to implement the NCR approach was vocally explained to the owners. In the second and third intervention phases (modelling, MOD, and modelling and feedback, MOD and FB, respectively), the first author’s demonstrations of the correct implementation of the NCR approach (modelling) and constructive feedback were applied. While the owners were free to use the intervention (NCR) in their daily interactions with the dogs, the data collection sessions were limited to a maximum of two sessions per day to prevent fatigue and satiation (repeated presentation of a reinforcer weakens the reinforcer’s effectiveness, which results in a decline of response rate; [33]).

##### Baseline

Counts of jumping up within the first minute of each dyads’ tangible or attention conditions (four or five cycles each) during the FA were used as baseline measures and were counted from video records of each dyads’ FAs. The rationale for omitting separate baseline measurements was to enable dyads to proceed directly into the first BST phase (oral instruction) after completing the elaborative FA stage (postponing any longer intervention on the problem behaviour).

##### Oral Instruction

The oral instruction phase started with the vocal presentation of the NCR training instruction. Therefore, the first author read respective instruction to the participants once (Table 4 shows instruction for tangible and attention type of reinforcement). Subsequently, participants had the opportunity to ask questions and clarify certain points. Once the owners stated that they had acquired all the necessary information to perform the training with their dogs, data collection commenced. As soon as training with the participating dogs was initiated, additional owner questions were solely met with “implement the instruction to the best of your knowledge” (no additional instructions were provided to the owners). This procedure was repeated at the onset of every new data collection day. A maximum of two sessions per day with a break of at least five minutes in between sessions was performed. During the delivery of the instruction and during the breaks, the owners remained outside the assessment area (separated from the dog) to prevent confounding, such as satiation.

If the owners performed with highest procedural integrity three times in a row (Step 6 or 100%), the experiment ended at this point for the respective dyad. Overall, a maximum of four sessions could be conducted for the oral instruction phase.

##### Modelling by the First Author

Prior to the first owner-led session of the modelling phase, the first author demonstrated and orally explained each step of the intervention. A “fake dog” (i.e., stuffed, life sized, black dog; Melissa and Doug^®^) was used for modelling and role play. The fake dog was animated by the owner, acting like a real dog, while the first author took the role of the owner, thereby modelling the procedure. Modelling was conducted in a separate room, away from the participating dog. This measure was implemented, as repeated use of the participating dogs during this phase could have confounded their performance during the actual training. After modelling by the first author was finished, the owners once again were given the opportunity to ask questions to clarify the procedure. During this question-and-answer step, uncertainties were answered, however, no further modelling or feedback on previous performances was provided. Modelling and answering questions took approximately five minutes. Once the owners started training their own dogs, additional questions were solely answered with “implement the instruction to the best of your knowledge” (no additional instructions were provided to the owners). Modelling was repeated at the onset of every new data collection day. A maximum of two sessions per day with a break of at least five minutes in between sessions could have been performed. During the breaks, the owners stayed outside the assessment area, separated from the dog to prevent confounding.

If the owners performed with highest procedural integrity three times in a row (Step 6 or 100%), the experiment ended at this point for respective dyad. Overall, a maximum of four sessions could be conducted during the modelling phase.

##### Modelling and Feedback

This last phase had the purpose of increasing owner and dog performance to criterion (i.e., 100% procedural integrity for owners, and dogs displaying three jumps or less on average across three consecutive sessions). It consisted of modelling and feedback using the same fake dog, and modelling was conducted in the same way as stated previously. However, the role-play part was extended by switching roles, i.e., the first author adopted the role of the dog, while the owner had the opportunity to rehearse the procedure. Feedback comprised approving statements of steps correctly performed and pointing out steps incorrectly performed during previous phases (oral instruction and modelling). Explanations on why identified steps were incorrect and modelling to illustrate how to perform them correctly were provided. This sequence was conducted once at every onset of a new data collection day. While owners could ask as many questions as necessary, the instructional part was restricted to 20 min. As with previous phases, once training of the actual dog was initiated, additional owner questions were solely answered with “implement the procedure to the best of your knowledge” (no additional instructions were provided to the owners). Modelling and feedback were conducted in a separate room, away from the participating dog. This was implemented because repeated use of the participating dogs during this phase could have confounded their performance during the actual training. A maximum of two sessions per day with a break of at least five minutes in between sessions was performed. During the breaks, the owners stayed outside the assessment area, separated from the dog, to prevent satiation.

Sessions continued until the owners and dogs reached the criterion (100% procedural integrity for owners, and dogs displaying three jumps on average across three consecutive sessions).

##### Generalisation

Generalisation sessions were introduced after owners’ and dogs’ behaviour reached criterion levels. For the generalisation test, the first author acted as the novel stimulus (less familiar person) and implemented the procedure in exactly the same way as during owner-conducted phases (NCR was delivered in 10 s intervals alternating with 10 s intervals of withholding reinforcement until 60 s have passed). Four sessions across two weeks (two sessions per appointment, once a week) were conducted to investigate any short-term generalisation effects. Count of jumping up per session and the first author’s procedural integrity were measured.

After the generalisation test, the owners were asked to implement the NCR procedure as often as possible. No further instructions were provided within the following three weeks.

##### Follow-Up Probe

Three weeks after the last generalisation session, one follow-up probe session was carried out. The owners were told to “do as you have previously done” and no additional information was provided. This appointment was conducted in the exact same manner as previous NCR sessions (NCR was delivered in 10 s intervals alternating with 10 s intervals of withholding reinforcement until 60 s have passed). Data on count of jumping up and owner procedural integrity were collected.

##### Social Validity

After conducting the follow-up session, participants were emailed a weblink and asked to complete the social validity questionnaire available online. This was done in an effort to establish the acceptability of the FA and training methods used (i.e., NCR and BST). Within this questionnaire, the owners had opportunities to give qualitative feedback and suggest improvements.

##### Procedural Integrity of the First Author

Data on the procedural integrity of the first author’s BST teaching skills were collected. For each BST phase (oral instruction, modelling, and modelling and feedback), customised intervention integrity checklists were used (adapted from Warrington [94]). The following items of the first author’s behaviour were recorded: (a) presentation of the oral instruction; (b) preparation of equipment (e.g., instructions, fake dog, tangibles); (c) inclusion of each of the five components as listed in Table 2 (Step 6); (d) delivery of modelling; (e) participation in role-play (acting as dog or owner); and (f) delivery of feedback based on modelling and role-play. Procedural integrity was assessed from the video recordings of sessions and was scored as percentage of above items presented or not (number of items presented divided by number of items presented plus items not presented multiplied by 100). Overall, researcher procedural integrity was 90.4% across all three BST phases and dyads (range, 88% to 93%).

### 4.2. Interobserver Agreement

#### 4.2.1. Count of Jumping Up and Owner Procedural Integrity of BST

Interobserver agreement (IOA) was calculated for count of jumping-up responses, 10 s ± 2 s intervals, and owners’ procedural integrity ranks (see Table 2 for task analysis). Agreement was assessed for 33% of the recorded sessions across all four dyads. Therefore, out of 42 video recordings, 14 videos were second coded (i.e., one session out of each intervention phase including follow-up probes). IOA was calculated by dividing agreements by the total number of agreements plus disagreements and multiplied by 100 ([13] p. 116). For BST ranks and counts of jumping up, an agreement was scored when both observers recorded the same rank or counted the same number of jumps within each session. If a disagreement in one of the four phases’ sessions occurred (e.g., count of jumping up in modelling and feedback phase of dyad D01), 25% were deducted yielding an IOA of 75% for this dyad. For the 10 s ± 2 s intervals, access to tangible or attention, as well as ignoring was compared. To record an agreement, each interval (i.e., 10 s ± 2 s) had to have the corresponding correct interval across both observers.

Mean IOA for counts of jumping up was 75% for D01, and 100% for all other dyads. Mean IOA for 10 s ± 2 s intervals across dyads was 100%, 96% (range, 83% to 100%), 100%, and 83% (range, 50% to 100%) for D01, D02, D03, and D05, respectively. Mean IOA for BST ranks was 100% across all dyads.

#### 4.2.2. Count of Jumping Up and First Author’s Procedural Integrity of Generalisation Phase

IOA was assessed for 25% of the video recordings (i.e., one recorded session for each dyad), and was calculated in the same way as above.

Mean IOA for counts of jumping up was 100% across all dyads. Mean IOA for 10 s ± 2 s intervals across all dyads was 92% (range, 83% to 100%). Mean IOA for BST ranks was 100% across all dyads.

### 4.3. DataAnalyses

Same as in Experiment 1, visual analysis and Tau calculations were implemented to determine the effectiveness of the NCR procedure (as recommended by Kratochwill et al. [76]; Parker et al. [77]). Please see Section 3.3 Data Analyses for details.

### 4.4. Results

#### 4.4.1. Effectiveness and Procedural Integrity

Table 6 shows averaged effect size values (i.e., averaged Tau) and corresponding statistical information of NCR intervention contrasted to baseline phase across each intervention phase (oral instruction, modelling, and modelling and feedback) per dyad. Tau and respective statistics for the NCR intervention during generalisation phase are separately presented, as this phase was conducted by the first author, while the other phases were led by the dog owners.

**Figure 2:**
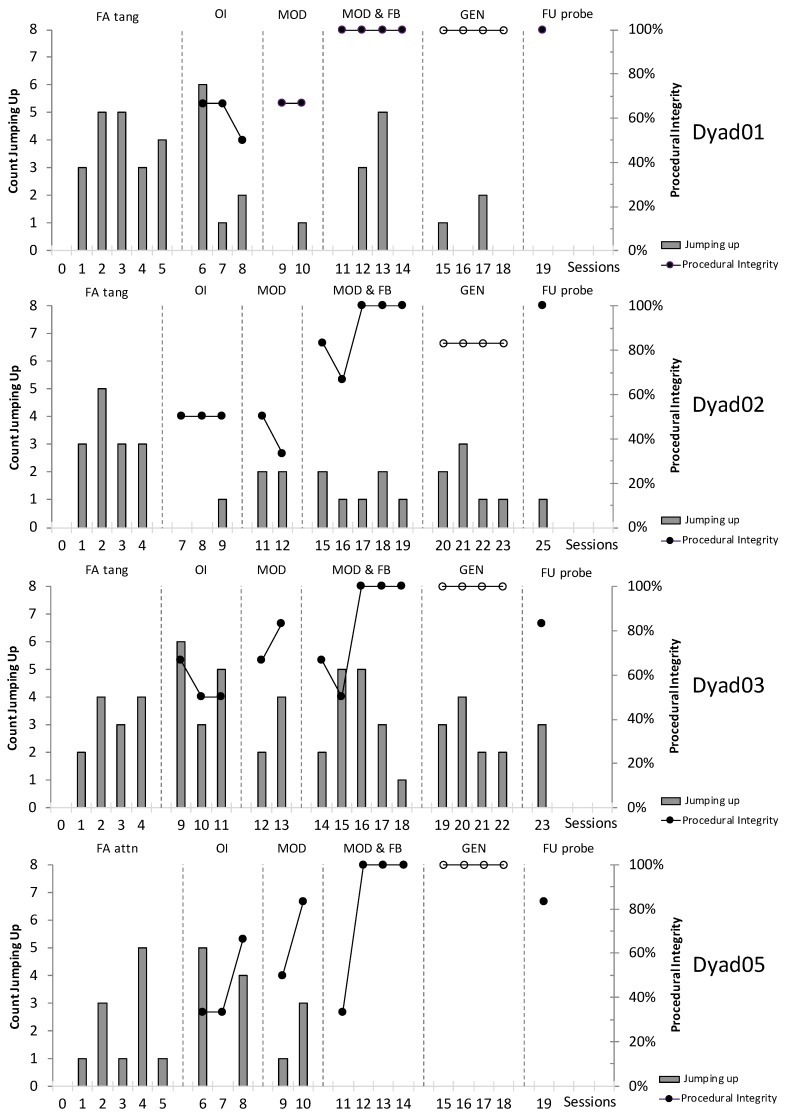
Count of dogs’ jumping-up responses (left y-axis) and procedural integrity of owners’ implementation of the NCR training, expressed in percentages, during different conditions of BST intervention (right y-axis). Data on owner procedural integrity are not shown during baselines (FA tang and FA attn for dyads 01 to 03 and dyad 05, respectively) because the BST intervention was not provided during these phases. Each horizontally arranged panel displays the indicated dyad’s data throughout weekly sessions. Each of the BST phases are presented in separate columns which are divided by dashed vertical lines. The phases labelled with “GEN” (“generalisation sessions”) were conducted by the experimenter. Respective procedural integrity data are depicted as open circles contrasting owner procedural integrity data points (closed circles). Data on FU probes were collected three weeks after the last GEN session. Note. FA tang/attn = functional analysis tangible/attention condition; OI = oral instruction; MOD = modelling; MOD and FB = modelling and feedback; GEN = generalisation sessions; FU = follow-up probe.

Figure 2 depicts count of jumping up and percent procedural integrity of dog owners and the first author for the NCR procedure and BST phases across all dyads. Overall, the NCR intervention yielded important decreases of jumping up in D01, D02, and D05, with mean percent decreases ranging from 25% to 64% (mean range 1.2 to 1.7 jumps per minute) when compared to pre-intervention means (mean range 2.2 to 4 jumps per minute). For D03 a small increase of 11% in jumping was found (mean range, 3.6 to 3.25 jumps per minute). Table 7 presents individual dyads’ results.

**Figure 3:**
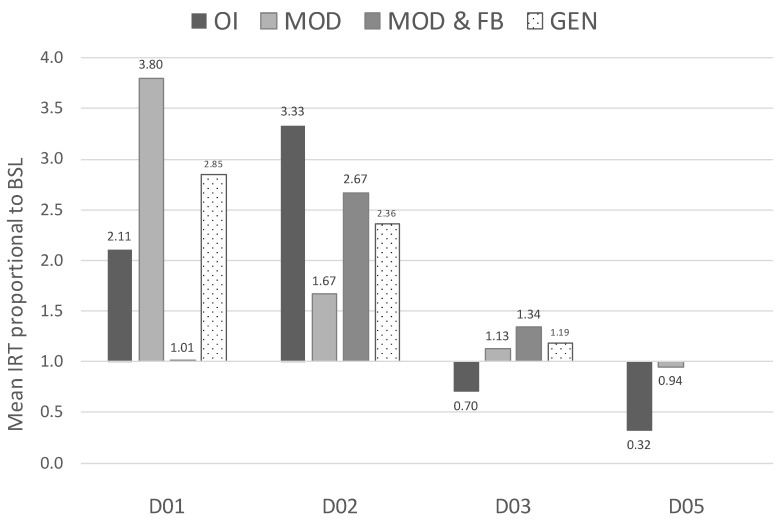
Mean inter-response times (IRT) proportional to baseline measures (FAtang or FAattn) across owner-led BST phases and the generalisation phase, which was carried out by the first author. Values below 1 represent shorter mean IRTs between jumps, when compared to baseline measures (increase in rate of responses). Proportions were calculated by averaging the IRT of each phase and dividing it by the average IRT of baseline. For example, in the case of dog D03 during OI phase, mean IRT of 14 s was divided by mean baseline IRT of 20 s, which resulted in a proportional IRT of 0.70. This value indicates that the length of IRTs dropped 70% when compared to baseline levels.

Figure 3 displays the dogs’ mean IRT proportional to the baseline measures (FAtang or FAattn) across owner-led BST phases and generalisation sessions conducted by the first author. Longer IRTs entailed instances of jumping up that were more separated across time. Higher proportions in Figure 3 indicate increased IRTs as compared to baseline. Dogs D01 and D02 had displayed increased IRTs in all conditions proportional to baseline, with the largest increase observed during MOD for D01 (almost four times above baseline—3.8) and OI for D02 (more than a threefold increase). Dog D03 showed more modest increments in IRTs across all conditions (smaller than twofold), except during OI in which a small reduction in IRTs was observed (indicated by the value being below 1—i.e., 0.70). Dog D05 showed same levels of IRTs for MOD, MOD and FB, and GEN (proportion approximately 1), and an important decrement in IRTs during OI (0.32).

Procedural integrity of all owners was between 33% and 83% across oral instruction and modelling phases, and was further improved during modelling and feedback phases, reaching criterion (three consecutive sessions with 100% integrity or Step 6) within five sessions across all owners. Table 8 displays individual results.

#### 4.4.2. Generalisation Phase

Table 9 displays mean jumps per minute, percent reduction of jumping up (compared to FA tang/attn baseline responding), and mean percent of the first author’s procedural integrity during generalisation phase. Overall, jumping up behaviour decreased in all four dogs, with D01 and D05 showing a reduction of >80% (81% and 100%, respectively). The first author’s mean procedural integrity was 96% (range, 83.3% to 100%) across all dogs.

#### 4.4.3. Follow-Up

Five weeks after the last modelling and feedback session (i.e., three weeks after the last generalisation session), one owner-led follow-up session was performed. Overall, jumping up decreased when follow-up measures were compared to baseline levels. The mean reduction across all four dyads was approximately 70% (range, 7.7% to 100%), with D03 showing the least reduction (3 jumps per minute compared to mean 3.25 jumps per minute during baseline). Dyad 02′s jumping up decreased by 71% (1 jump per minute compared to mean 3.5 jumps per minute). Both D01 and D05 showed a decrease by 100%, reducing jumping up to zero occurrences during follow-up probes.

Overall, owner procedural integrity continued to stay high after five weeks of no BST instruction. Owners’ procedural integrity with the NCR procedure was 83% for D03 and D05, while owners of D01 and D02 continued to perform with 100% integrity.

#### 4.4.4. Social Validity

Owners rated the effectiveness and acceptability of the procedures on a 5-point Likert-type scale (1 = strongly disagree, 5 = strongly agree). Table 10 displays the 10 questionnaire items, as well as respective ranks chosen by the owners, and respective means and standard deviations (SD). All four owners stated that they liked the methods used (Item 10), however, all of them also stated that they think oral instruction would have been sufficient to train their dogs according to the specifications (Item 1). Items regarding the effectiveness of the other two BST components (modelling and modelling and feedback; Items 2 to 4) were answered with high agreement ranks (Ranks 4 and 5). Generally, owners found BST effective and helpful (Item 5). Owners also agreed and strongly agreed on the efficacy, helpfulness and harmlessness of the NCR training approach (Items 5 to 8).

### 4.5. Discussion

Experiment 2 demonstrated that NCR effectively reduced jumping up behaviour in three out of four dogs (dogs D01, D02, and D05). This is in line with findings from human-related research. Several studies found that NCR was effective in reducing challenging behaviours across a broad range of ages (e.g., 3 to 48 years; [95,96]), response topographies (e.g., aggression, coprophagia, saliva play, self-injurious behaviour; [97,98,99]), settings (e.g., participants’ homes or schools; [100,101]), and functional characteristics of behaviours (e.g., social or automatic reinforcement; [71,102]). Results of recent meta-analyses by Richman et al. [102] and Ritter, Barnard-Brak, Richman, and Grubb [103], further support the effectiveness of NCR on the reduction of challenging behaviour in human learners. Effect size analyses indicated a large effect (e.g., Cohen’s *d* = −1.58; [102]) for reduction of problem behaviours or, put differently, NCR procedures accounted for 60% of the problem variance between baseline and treatment phase [102].

Notwithstanding that NCR is an empirically supported treatment according to the criteria put forward by Horner and Kratochwill [104] and Kratochwill, Hitchcock, Horner, Levin, Odom, Rindskopf, and Shadish [105] (e.g., a practice is considered evidence-based when repeated and convincing documentation of functional relations between introduction and change in valued outcomes exists), incorporation of NCR as an intervention in applied animal-related studies has been slow (e.g., [106]). While the present intervention yielded promising results with three dogs (dogs D01, D02, and D05), the behaviour of one dog did not respond to the NCR intervention (dog D03). These findings were supported by effect size calculations to determine the efficacy of NCR across all four dyads. The range of effects of the NCR intervention were small to very large (e.g., Tau 0.15 and Tau −1, CI 90% [−0.29 – 0.61] and [−1 – −0.55], *p* = 0.569 and 0.0009 for D03 and D02, respectively), with negative algebraic signs indicating a reductive effect of the intervention.

Although the identified functional social reinforcer (Experiment 1) was used for the intervention with dog D03 (access to tangible delivered by the owner), the rate of jumping remained around baseline level (three jumps per minute). This is contrary to the notion that using the functionally identified reinforcer during the NCR intervention, has a desired impact on the effectiveness of NCR procedures, namely, a decrease of undesired behaviour [102]. One possible reason the behaviour of dog D03 did not change in the anticipated way, relates to the time-interval chosen [69,107]. Previous studies have shown that extended schedules of reinforcement in NCR interventions resulted in increases in undesired responding in human learners [71,102]. It is possible that the 10 s intervals between response-independent reinforcement were too wide for this individual dog. Possibly, this particular dog might have benefitted from a denser schedule (e.g., 5 s intervals), and implementing subsequent schedule thinning (gradual extension of time intervals between access to reinforcement; [108]). Future research could possibly benefit from establishing the intervals for each dog by using baseline rate of jumping (e.g., for high levels of jumping).

To summarize, the findings from the current experiment show that NCR implemented in accordance with an individualised, function-based approach (use of functional reinforcers), yielded important decreases in jumping-up behaviour in the majority of dogs. These results are promising as they indicate that NCR may be effective in companion dogs. However, results are tentative, and more research is needed to investigate the effectiveness and generality of the current findings.

With regard to BST, Experiment 2 also showed that BST was successful in teaching owners to perform a dog training intervention with high fidelity. During the oral instruction phase, procedural integrity of the owners was about chance level, and it did not reach criterion during the modelling phase. However, all owners reached criterion within a relatively short amount of time during the modelling and feedback phase (four to five sessions, which corresponds to a maximum of approximately 60 min of teaching and training). This may be an important feature of BST for clinical application, as owners may require less time to learn a new skill with the help of BST. However, since time to criterion was not directly measured in this experiment, future studies could analyse this variable.

Owner criterion levels were only obtained after all three BST phases were presented. Since the phases were introduced consecutively, the effect of each individual phase is inseparable from those effects resulting from preceding phases (sequence effects; [13,68]). Future studies using BST could perform component analyses (systematic analyses of two or more independent variables–components–comprising an intervention package; [109]) to assess the necessary and active components of BST (e.g., [110]). Nonetheless, the results demonstrate the effectiveness of BST as a package to train dog owners on the implementation of NCR.

With the implementation of the generalisation phase, we aimed at investigating whether the dogs’ newly acquired skills would generalise across a new person. Contrary to conducting only one or two generalisation probes, we were interested in the dogs’ behaviour change across time. Hence, the generalisation phase was carried out across two weeks. As per definition, which states that none or limited extra training is needed to observe behaviour change across time, novel people or settings [39,111], results showed that without prior experience and additional training with the first author, jumping up responses stayed low in all four dogs. These results are in line with the findings of a previous study [18], that reported generalisation of dogs’ skills (e.g., “sit” and “wait”) across novel volunteer trainers in a shelter setting. The current experiment, however, did not test generalisation across a second novel person who may not have had the first author’s knowledge about the NCR procedure.

The results of the follow-up probe sessions showed that owners retained the acquired skills over five weeks, and that they could implement the NCR procedure with high fidelity. These findings are consistent with human-related BST studies which also reported that caregivers were able to display the newly acquired skills with high treatment fidelity several weeks after the training phases ceased (e.g., [65,112]).

To summarise, the current BST procedure yielded both generalisation of dog behaviour and response maintenance of correct owner performance and behaviour change in dogs. High integrity owner performance during maintenance probes suggest that BST may be helpful in increasing the likelihood that owners continue to use the intervention without ongoing feedback from the animal behaviourist.

## 5. General Discussion and Conclusions

This study found that owner-led FAs are feasible and yield valid results for assessing the function of jumping up behaviour in companion dogs. Further, it showed that teaching the owners how to implement an intervention with high integrity can be achieved by using BST. The intervention taught, namely NCR, effectively decreased jumping up in three out of four dogs.

Experiment 1 found that reinforcers maintaining jumping up behaviour in dogs were mostly related to accessing tangibles (dogs D01, D02, D03, and D04), while jumping up was reinforced by owner attention in only one dyad (D05). This is consistent with previous research using FAs with companion dogs. Dorey et al. [59], for example, reported that jumping up was maintained by either access to a tangible or attention in two and one dogs, respectively. Several studies reported that FAs were helpful in identifying the reinforcers that maintained undesired behaviour, and that these functional reinforcers could be successfully used to change the dogs’ behaviours [24,57,59]. However, Experiment 1 adds an important aspect to the existing literature. Namely, that FAs can be carried out by dog owners, yielding meaningful outcomes. Taken together, these findings may hopefully lead to the more widespread adoption of FAs into the clinical animal behaviour practice. However, further research is warranted to expand the knowledge about if and how conditions (e.g., attention, control/play, demand, ignore, and tangible) can be adjusted to better suit the requirements of individual clients in particular, and the clinical animal behaviour practice in general [24]. For example, conditions may be added (e.g., alone—leaving the dog unaccompanied while video recording his or her behaviour for the duration of a session; [57]), or modified according to level, trend and variability in data (e.g., inconclusive results; [24]).

Despite the advantages of implementing FAs, i.e., experimentally establishing functional relations and their inherent flexibility (tailored to the individual settings of the learners; [36]), FAs should be carried out only provided certain criteria are met. First, even if FAs can contribute to ruling out potential underlying medical issues, medical consultation with a veterinary practitioner should be sought first if there is any reason to believe that the problem behaviour is influenced by physiological variables (e.g., pain or hormonal imbalances; [113]). Therefore, in cases where an underlying medical cause is suspected, animal behaviour professionals should recommend medical examination of the animal prior to the onset of the FA and respective intervention [113,114,115]. Second, not all problem behaviours may be equally suitable for conducting FAs. Although it may be feasible to safely conduct FAs (e.g., protected contact and muzzling the dog) with higher ranking aggressive responses toward humans or other animals (e.g., snapping and/or biting; [116]), the use of FAs should be carefully evaluated and only applied based on animal welfare considerations. Observations involving descriptive functional assessments (e.g., ABC assessment) and/or interviews of the owners may be more appropriate options for determining the function of severe problematic canine behaviour. Both aspects require comprehension of specifics of canine behaviour, as well as underlying behavioural processes and understanding of the concepts and principles of behaviour analysis as they relate to designing interventions unique to each client and learner [114].

Experiment 2 showed that BST is useful to teach dog owners the implementation of an intervention resulting in high procedural integrity. While the importance of treatment integrity for intervention success (e.g., increase in desired behaviour or decrease in undesired behaviour; [13]) has been demonstrated and discussed in several studies (e.g., [117,118,119]), the current experiment supports this notion only tentatively. For example, although a decrease in counts of jumping up was detected from the onset of the intervention for dyad D02, and despite owner procedural integrity increasing during the modelling and feedback phase, jumping up decreased only slightly during this last phase. One reason for the weak correspondence between procedural integrity and decrease in undesired canine behaviour may be found in the NCR intervention. Fixed-time NCR procedures have been reported to be relatively easy to implement when compared to differential reinforcement approaches ([89,120,121]). With contingency-based interventions, caregivers (parents or owners) need the skills to identify the alternative behaviour and provide reinforcement contingent on the desired responses with high treatment integrity (e.g., technique-focused caregiver-mediated interventions; [122]). These skills are not required for response-independent time-based reinforcement strategies (i.e., NCR). Future research should determine the correspondence of procedural integrity with response-contingent reinforcement, as well as NCR with owner–dog dyads.

Experiment 2 further demonstrated that NCR effectively reduced jumping up behaviour in three out of four dogs. This is in line with previous human-related studies, which have shown that NCR interventions are feasible and effective across a wide range of undesired behaviours (e.g., aggression, disruption, inappropriate speech, pica, rumination, and stereotypic behaviour; [73,89,102]). Although its clinical utility with human learners has been repeatedly demonstrated, the underlying behavioural processes of NCR still have not been confirmed [90]. The two mechanisms commonly cited as being responsible for NCR’s effectiveness are operant extinction and satiation [63,90]. Operant extinction is defined as withholding reinforcement after a response occurred, therefore, cancelling the reinforcement contingency [123]. With NCR, the contingency between the response and the reinforcer is disrupted because the reinforcer is presented on a response-independent time-based schedule [90]. Satiation is defined as the repeated presentation of a reinforcer which decreases its reinforcing properties [33]. Satiation may be responsible for reductions in responding because noncontingent access to the reinforcer eliminates the motivation to engage in the undesired behaviour [89] (i.e., an abolishing operation, which has an abative effect on behaviour [13]). Based on the immediate reduction of jumping up responses during the NCR intervention across three dogs (D01, D02, and D05; [124]), it is hypothesised that satiation may have been the main mechanism. However, it is likely that NCR achieves its efficacy through a combination of processes [89], and that these processes vary across individuals. Future canine-related studies should investigate the underlying behavioural processes and respective conditions under which the different mechanisms may occur [89]. Additionally, further research could assess different schedules of reinforcement with regard to NCR’s efficacy (e.g., fixed-time versus variable-time schedules; [69]).

The current study has several limitations. First, the sample size of five participants in Experiment 1, and four during Experiment 2 may be considered a limitation. However, from a SCRM point of view, the number of participants is appropriate for the purpose (feasibility and efficacy of methods) and respective research designs (multielement design and changing conditions design; [93]). From the SCRM perspective, each dog is an experimental systematic replication on its own (i.e., analysis is at the level of the individual and improvement of one individual’s challenging behaviour is meaningful), with each dyad experiencing all the baseline and treatment conditions [125,126]. Nonetheless, from this perspective it is also assumed that more systematic replications involving not only larger numbers of participants reliably showing the promising effects, but also other settings, problem behaviours, refinements of the interventions, etc. are warranted to improve validity and increase generality [127]. Second, the use of edibles during the demand condition of the FA may have had an impact on dogs’ D01 and D03 jumping up behaviour. Food can be a powerful reinforcer for dogs [24]. Hence, its presence and use could have resulted in a confounding effect by acting as an MO for jumping up (due to typically occurring mild food deprivation of the dogs as data collection was scheduled prior to their feeding times). Future research should investigate the use of food items during FA conditions with dogs (e.g., during demand or control conditions). Third, we did not conduct separate baseline measures prior to the onset of the BST intervention. This prohibited comparing the effects BST had on the owners’ procedural integrity data prior to the initiation of BST. Subsequent studies should conduct baseline measurement to get a clearer picture of the skills dog owners may already be equipped with prior to the implementation of a behaviour change programme (e.g., “experience”; [1]). Fourth, the modelling phase comprised only two data points. This limits the validity of this phase as stated by the “Single-Case Design Technical Documentation” standard which recommends a minimum of three data points per phase to qualify as an attempt to demonstrate an effect [128]. Hence, future research should test in more detail the effects of the modelling phase and other components on owners’ procedural integrity (e.g., component analysis; [109]).

In conclusion, Experiment 1 successfully identified reinforcers maintaining the behaviour of jumping up in all five dyads. For the majority of dogs, access to a tangible item was the maintaining reinforcer (dogs D01, D02, D03, and D04). Only for dog D05, owner-provided vocal and tactile attention was maintaining the jumping up responses. Experiment 2 demonstrated that training dog owners to implement NCR effectively via BST reduced jumping up behaviour in three out of four dogs (dogs D01, D02, and D05). Although promising, these results should be considered tentative, as more canine-related research into FA and NCR, as well as implementation of BST with animal caregivers, is needed. In this vein, future research should also investigate the utility of the approaches advocated here for other problem behaviours. Such behaviours may include, but are not limited to, excessive barking, responses related to overexcitement and/or stereotypic behaviours (e.g., mounting or spinning). It is hoped that in the future ABA’s inherently experimental and individually tailored approaches (e.g., continuous measurement of target behaviour by means of SCRMs, functional behaviour assessments, and emphasis on reinforcement), are more readily adopted into the field of clinical animal behaviour.

## Figures and Tables

**Table 1:** Overview of participating dyads’ details (dyad identification, breed, age, sex and owner information), as well as cued requests, type of treats used as edible reinforcers, and type of items (tangible) used during functional analyses. Note: m = male; f = female; i = intact; n = neutered.

Dyad	Breed	Age	Sex	Owner	Request	Edible	Tangible
D01	Cocker Spaniel	3 years	m/i	Female (31–40 years) Two dogs	Repertoire: sit, hand touch New: weaving through legs	Dry food	Tennis ball
D02	Mixed breed	4 years	f/n	Female (31–40 years) First dog	Repertoire: sit, down New: beg	Dry food	Large ball
D03	Dutch Shepherd	4 years	m/n	Female (21–30 years) Multiple dogs	Repertoire: sit, hand touch New: bow	Dry food	Plush toy
D04	Miniature Poodle	4 years	f/i	Female (51–60 years) Multiple dogs	Repertoire: sit, down New: hand touch	Dried Meat stripes	Rubber ball
D05	Mixed breed	12 years	f/n	Female (51–60 years) Multiple dogs	Repertoire: sit, down New: paw	Pieces of cheese	Leash and leaving ^1^

^1^ For dog D05 the owner identified leashing and leaving the house as a reinforcer. It is herein assumed as a tangible as suggested by Dorey et al. [59].

**Table 2:** Summary of cycles completed and number of sessions per dyad.

Dyads	Cycles Completed	Number of Sessions
D01	5	25
D02	4	20
D03	4	20
D04	10	50
D05	5	25

**Table 3:** Tau calculations and respective statistical information across contrasted conditions (i.e., numbers 1 to 9) are provided for dyad D01. Combined conditions and averaged statistics for attention and tangible conditions are shown at the bottom of the table (i.e., numbers 10 and 11). Positive Tau values indicate an increase in jumping up during the contrasted condition as can be seen in Number 5 (i.e., Tau 0.2, CI 90% [−0.43–0.83], *p* = 0.6015), for example. Negative values indicate a decrease in jumping (e.g., Number 6, Tau −0.36, CI 90% [−0.99–0.27], *p* = 0.3472). Statistically significant comparisons are identified by an asterisk (*).

Sequence of Calculation	Contrasted Conditions	Tau	CI 90%	*p*-Values
1	control vs. attention	0.72	0.09 to 1	0.0601
2	control vs. demand	1	0.37 to 1	0.009 *
3	control vs. ignore	0.72	0.09 to 1	0.601
4	control vs. tangible	1	0.37 to 1	0.009 *
5	attention vs. tangible	0.2	−0.43 to 0.83	0.6015
6	attention vs. demand	−0.36	−0.99 to 0.27	0.3472
7	attention vs. ignore	−0.4	−1 to 0.23	0.2963
8	tangible vs. demand	1	0.37 to 1	0.009 *
9	tangible vs. ignore	1	0.37 to 1	0.009 *
**Averaged**				
10	5 + 6 + 7 (all attention)	−0.19	−0.55 to 0.18	0.3985
11	5 + 8 + 9 (all tangible)	0.73	0.37 to 1	0.0009 *

**Table 4:** Oral instructions used during respective BST phase. The left column displays the instruction for dyads using a tangible (i.e., toy) as reinforcer, and the right column provides the instruction for dyad 05 who used vocal and tactile attention as reinforcement.

Oral Instruction	
Tangible Reinforcement	Attention Reinforcement
While walking through the door, lower your upper body and hold the toy towards the floor. Immediately on entering, let your dog interact with the toy for 10 s. When 10 s have passed, take the toy from your dog, and do not look at him/her for 10 s. Hold the toy in a manner that is comfortable for you and so the dog cannot access it. After 10 s, return the toy to your dog and let him/her engage with it for another 10 s. This sequence is repeated three times until 60 s have passed, irrespective of your dog’s behaviour. Once the sequence has been repeated three times, you leave the room, taking the toy with you. To ensure that you keep to the 10 s time intervals, please count from 21 to 30 during each interval.	While walking through the door, lower your upper body, with your hands loosely reaching towards the floor. Provide attention to your dog by talking to her in a soft tone and/or gently stroke her for 10 s. After 10 s has passed, stop attending to your dog and do not look at her for 10 s. Irrespective of your dog’s behaviour, pet and/or talk to her again for another 10 s. This sequence is repeated three times until 60 s have been completed. Once the sequence has been repeated three times, you leave the room. To ensure that you keep to the 10 s time interval, please count from 21 to 30 during each interval.

**Table 5:** Procedural integrity of NCR training procedure. The list is displayed in hierarchical order from fully correct (Step 6) to incorrect (Step 0). The hierarchical steps were used to define and measure owners’ procedural integrity with the NCR procedure.

Steps	Description of Each Step
	Attention/Tangible Reinforcement
6	Bending over, with hands towards the floor when entering. A soft voice was used during the attention intervals (10 s ± 2 s). Not looking at the dog and standing still for 10 s ± 2 s was implemented. The sequence was repeated until 60 s ± 6 s have passed (6 intervals), then the owner left the training area through the front door.
5	Bending over, with hands towards the floor when entering. A soft voice was used during the attention intervals (10 s ± 2 s). Not looking at the dog and standing still for 10 s ± 2 s was implemented. The sequence was correctly repeated twice. The owner left the training area through the front door.
4	Bending over, with hands towards the floor when entering. A soft voice was used during the attention intervals (10 s ± 2 s). Not looking at the dog and standing still for 10 s ± 2 s was implemented. The sequence was correctly repeated once. The owner left the training area through the front door.
3	Standing upright, with hands reaching towards the floor when entering. Attention or ignore responses were either too short or too long (<10 s ± 2 s>) across two intervals. A soft voice was used during the attention intervals. Not looking at the dog and standing still for 10 s ± 2 s did not occur in at least one of the sequences. The owner left the training area through the front door.
2	Standing upright, with hands held at hip height when entering. Attention or ignore responses were either too short or too long (<10 s ± 2 s>) across three intervals. A high-pitched voice was used during the attention intervals. Not looking at the dog and standing still for 10 s did not occur in at least two of the sequences. The owner left the training area through the front door.
1	Bending over, with hands toward the floor when entering. Attention was continuously provided across all 10 s ± 2 s intervals. The owner left the training area through the front door.
0	Standing upright, with hands toward the floor when entering. Attention was not provided or undesired attention (i.e., reprimanding or scolding of the dog; vocal attention only) was given. The owner did or did not leave the training area through the front door.

**Table 6:** Effect sizes (Tau) and corresponding statistical values (CI and *p*-values) for the NCR intervention averaged across BST phases (i.e., averaged Tau; oral instruction, modelling, and modelling and feedback) are displayed per dyads (D01, D02, D03, and D05). Statistics for the contrast of baseline (BSL) vs. generalisation phase (GEN) are provided separately, as this phase was conducted by the first author (as indicated by open dots °). Statistically significant results (i.e., *p* < 0.05) are indicated by asterisks (*).

	Statistics	D01	D02	D03	D05
Averaged Tau	Tau	−0.59	−1	0.15	−0.32
	CI 90%	−1 to 0.15	−1 to −0.55	−0.29 to 0.61	−0.76 to 0.11
	*p*-value	0.026 *	0.0003 *	0.569	0.220
BSL vs. GEN °	Tau	−1	−0.81	−0.37	−1
	CI 90%	−1 to −0.328	−1 to −0.100	−1 to 0.400	−1 to −0.32
	*p*-value	0.014 *	0.061	0.470	0.014 *

**Table 7:** Individual dyads’ mean rate of jumps per minute and percent reduction of jumping up displayed by baselines (FAtang and FAattn) and BST phases. Percent reduction was calculated as 1 minus mean jumps per minute divided by mean baseline jumps per minute multiplied by 100. Negative percentages (e.g., −43.6% in dyad 03) represent an increase in responding.

Dyads and Phases	Mean Jumps Per Minute	Reduction of Jumping ^1^
Dyad 01		
Baseline	4	–
Oral instruction	3	25%
Modelling	0.5	87.5%
Modelling and feedback	2	50%
Average (OI, MOD, MOD, and FB)	1.8	54.2%
Dyad 02		
Baseline	3.5	–
Oral instruction	0.3	90.5%
Modelling	2	42.9%
Modelling and feedback	1.4	60%
Average (OI, MOD, MOD, and FB)	1.2	64.4%
Dyad 03		
Baseline	3.25	–
Oral instruction	4.7	−43.6%
Modelling	3	7.7%
Modelling and feedback	3.2	1.5%
Average (OI, MOD, MOD, and FB)	3.6	−11.5%
Dyad 05		
Baseline	2.2	–
Oral instruction	3	−36.4%
Modelling	2	9.1%
Modelling and feedback	0	100%
Average (OI, MOD, MOD, and FB)	1.7	24.2

^1^ Compared to baseline responding.

**Table 8:** Individual owners’ procedural integrity displayed by BST phases.

Owners	Oral Instruction	Modelling	Modelling and Feedback
Owner dyad 01	61.1%	66.7%	100%
Owner dyad 02	50%	41.7%	90%
Owner dyad 03	55.6%	75%	83.3%
Owner dyad 05	44.4%	66.7%	83.3%

**Table 9:** Generalisation phase data conducted by the first author displayed by individual dogs.

Dyads	Mean Jumps Per Minute	Mean Percent Reduction of Jumps	Mean Procedural Integrity
Dog dyad 01	0.75	81.25%	100%
Dog dyad 02	1.75	50%	83.3%
Dog dyad 03	2.75	15.4%	100%
Dog dyad 05	0	100%	100%

**Table 10:** Social validity questionnaire featuring ranks chosen by each of the four owners and respective means and SD.

Rank Items	1—Strongly Disagree	2—Disagree	3—Partly Agree	4—Agree	5—Strongly Agree	Mean (SD)
1. I think that oral instruction by the researcher was sufficient to train my dog according to instructions.	–	–	–	–	4	5 (0)
2. I think that modelling using the fake dog was helpful to train my dog according to instructions.	–	–	1	2	1	4 (0.6)
3. I think that modelling and feedback including the use of the fake dog were helpful to train my dog according to instructions.	–	–	–	1	3	4.75 (1.9)
4. I think that the researchers feedback based on my training with the dog was effective and helpful.	–	–	–	1	3	4.75 (1.9)
5. I think that the Behavioural Skills Training method was effective and helpful to learn the skills necessary to train my dog.	–	–	–	3	1	4.25 (1.2)
6. I think that the time-based reinforcement approach was effective and helpful for training my dog.	–	–	–	2	2	4.5 (0.4)
7. I think that the time-based reinforcement approach was unproblematic for me and my dog.	–	–	–	1	3	4.75 (1.9)
8. I think that the time-based reinforcement approach was stressful for my dog.	3	–	1	–	–	(0.4)
9. Occurrences of jumping up decreased since initiating the intervention and training.	–	–	1	2	1	4 (0.6)
10. I like the methods used.	–	–	–	–	4	5 (0)

## Supplementary Materials

The following are available online at https://www.mdpi.com/2076-2615/9/12/1091/s1, Table S1: Tau calculations for functional analyses.
